# Adipokines as New Biomarkers of Immune Recovery: Apelin Receptor, RBP4 and ZAG Are Related to CD4^+^ T-Cell Reconstitution in PLHIV on Suppressive Antiretroviral Therapy

**DOI:** 10.3390/ijms23042202

**Published:** 2022-02-17

**Authors:** Elena Yeregui, Jenifer Masip, Consuelo Viladés, Pere Domingo, Yolanda M. Pacheco, Julià Blanco, Josep Mallolas, Verónica Alba, Montserrat Vargas, Graciano García-Pardo, Eugènia Negredo, Montserrat Olona, Judit Vidal-González, Maria Peraire, Anna Martí, Laia Reverté, Fréderic Gómez-Bertomeu, Manuel Leal, Francesc Vidal, Joaquim Peraire, Anna Rull

**Affiliations:** 1 Infection and Immunity Research Group (INIM), Hospital Universitari de Tarragona Joan XXIII (HJ23), 43005 Tarragona, Spain; elena.yeregui@iispv.cat (E.Y.); jenifer.masip@urv.cat (J.M.); cvilades.hj23.ics@gencat.cat (C.V.); veronica.alba@urv.cat (V.A.); mvargas.hj23.ics@gencat.cat (M.V.); ggarciap.hj23.ics@gencat.cat (G.G.-P.); molona.hj23.ics@gencat.cat (M.O.); anna.marti@iispv.cat (A.M.); laia.reverte@iispv.cat (L.R.); ffgomez.hj23.ics@gencat.cat (F.G.-B.); jperaire@comt.es (J.P.); 2 Institut Investigació Sanitària Pere Virgili (IISPV), 43005 Tarragona, Spain; 3 Infection and Immunity Research Group (INIM), Universitat Rovira i Virgili (URV), 43003 Tarragona, Spain; mperairelores@gmail.com; 4 CIBER Enfermedades Infecciosas (CIBERINFEC), Instituto de Salud Carlos III, 28029 Madrid, Spain; jblanco@irsicaixa.es (J.B.); mallolas@clinic.cat (J.M.); enegredo@flsida.org (E.N.); 5 Infectious Diseases Unit, Hospital de la Santa Creu i Sant Pau, 08025 Barcelona, Spain; pdomingo@santpau.cat; 6 Laboratory of Immunology, Institute of Biomedicine of Seville, IBiS, 41013 Seville, Spain; ypacheco-ibis@us.es; 7 UGC Clinical Laboratories, Virgen del Rocío University Hospital/CSIC/University of Seville, 41013 Seville, Spain; 8 IrsiCaixa AIDS Research Institute, 08916 Badalona, Spain; 9 Germans Trias i Pujol Research Institute (IGTP), Can Ruti Campus, 08916 Badalona, Spain; 10 Infectious Diseases and Immunity, Faculty of Medicine, Universitat de Vic-Universitat Central de Catalunya (UVic-UCC), 08500 Vic, Spain; 11 HIV Unit and Infectious Diseases Service, Hospital Clinic-IDIBAPS, 08036 Barcelona, Spain; 12 Fundació de la Lluita contra les Infeccions, Hospital Universitari Germans Trias i Pujol, 08916 Badalona, Spain; 13 Faculty of Medicine, Universitat de Barcelona (UB), 08007 Barcelona, Spain; judit.vidal.gonzalez@gmail.com; 14 Internal Medicine Service, Hospital Viamed Santa Ángela de la Cruz, 41014 Seville, Spain; mleal@telefonica.net

**Keywords:** adipokines, antiretroviral therapy, HIV, immunodiscordant response, poor immune recovery

## Abstract

A significant proportion of people living with HIV (PLHIV) who successfully achieve virological suppression fail to recover CD4^+^ T-cell counts. Since adipose tissue has been discovered as a key immune organ, this study aimed to assess the role of adipokines in the HIV immunodiscordant response. This is a multicenter prospective study including 221 PLHIV starting the first antiretroviral therapy (ART) and classified according to baseline CD4^+^ T-cell counts/µL (controls > 200 cells/µL and cases ≤ 200 cells/µL). Immune failure recovery was considered when cases did not reach more than 250 CD4^+^ T cells/µL at 144 weeks (immunological nonresponders, INR). Circulating adipokine concentrations were longitudinally measured using enzyme-linked immunosorbent assays. At baseline, apelin receptor (APLNR) and zinc-alpha-2-glycoprotein (ZAG) concentrations were significantly lower in INRs than in immunological responders (*p* = 0.043 and *p* = 0.034), and they remained lower during all ART follow-up visits (*p* = 0.044 and *p* = 0.028 for APLNR, *p* = 0.038 and *p* = 0.010 for ZAG, at 48 and 144 weeks, respectively). ZAG levels positively correlated with retinol-binding protein 4 (RBP4) levels (*p* < 0.01), and low circulating RBP4 concentrations were related to a low CD4^+^ T-cell gain (*p* = 0.018 and *p* = 0.039 at 48 and 144 weeks, respectively). Multiple regression adjusted for clinical variables and adipokine concentrations confirmed both low APLNR and RBP4 as independent predictors for CD4^+^ T cells at 144 weeks (*p* < 0.001). In conclusion, low APLNR and RBP4 concentrations were associated with poor immune recovery in treated PLHIV and could be considered predictive biomarkers of a discordant immunological response.

## 1. Introduction

Since the introduction of effective antiretroviral therapy (ART), the morbidity and mortality of people who live with HIV infection (PLHIV) has drastically decreased. However, up to 25–30% of PLHIV receiving ART when they are severely immunosuppressed fail to recover their CD4^+^ T cell counts, although they achieve correct viral suppression (VS) [1,2,3]. These individuals are known as “immunological nonresponders” or “immunological nonrecoverers” (INRs) and have a worse prognosis and a higher frequency of non-AIDS events [3,4,5,6,7,8]. Several factors have been traditionally linked to this failure, such as age, male sex, drug consumption, nadir CD4^+^ T cell counts, late ART introduction, or cytokine storm [1,2,3]. Nevertheless, the specific mechanisms that fully explain immune dysregulation in PLHIV are still unknown, and investigations of the molecular pathways preceding ART-associated poor immune recovery are needed to develop a useful tool for early detection and to ameliorate disease progression.

Recently, interest in adipose tissue and its role in regulating immune function has increased. Adipose tissue has been discovered as a relevant immune organ [9,10,11], and it may be involved in immune function and immune recovery in PLHIV receiving ART [12]. For instance, in previous years, several investigations have been conducted regarding adipose tissue and its role in HIV, concretely in ART-induced lipodystrophy and metabolic disturbances [13,14,15,16]. Both HIV infection and ART modify the adipose tissue composition, distribution, and function [12,17]. The virus itself targets adipose tissue directly by infecting adipose tissue-resident cells (notably CD4^+^ T cells) and indirectly via viral protein release, activation of inflammation, and the immune system and gut disruption [18]. Furthermore, in obese PLHIV, adipose dysfunction is boosted by inflammation, which in turn is an outcome of excess adipose and obesity-induced metabolic disturbances. In obese adipose tissue, the primarily immunoregulatory macrophages (M2 phenotype) increase to over 50% of total adipose tissue and shift to proinflammatory phenotype (M1), a contributing factor to the release of inflammatory cytokines [19]. In fact, among PLHIV, the activation of M1 phenotype is the primary source of low-grade chronic inflammation in the adipose tissue contributing to HIV-associated lipoatrophy [20]. ART introduction mitigates all these inflammatory processes but also contributes to increasing the adipose tissue dysfunction [21]. Indeed, adipose tissue has been advanced as a potential HIV reservoir, and thus viral persistence in adipose tissue after ART initiation might be related to the metabolic and immune dysfunctions of adipose tissue cells [22,23]. In this regard, we hypothesized that the secretion of adipokines, hormones with immunomodulatory effects secreted by adipocytes [10,24,25,26,27], may be related to CD4^+^ T cell depletion and restoration in PLHIV. Thus, in this study, we evaluated circulating concentrations of adiponectin, apelin, apelin receptor (APLNR), fatty acid-binding protein (FABP4), leptin, omentin, retinol-binding protein 4 (RBP4), resistin, vaspin, and zinc-alpha-2-glycoprotein (ZAG, also known AZGP1) in samples of ART-naïve patients starting ART and during follow-up at 48 and 144 weeks of ART. We aimed to relate plasma adipokine concentrations to nadir CD4^+^ T cell counts and to anticipate poor immune recovery using circulating adipokines as potential biomarkers.

## 2. Results

### 2.1. Patient Characteristics

**Figure 1:**
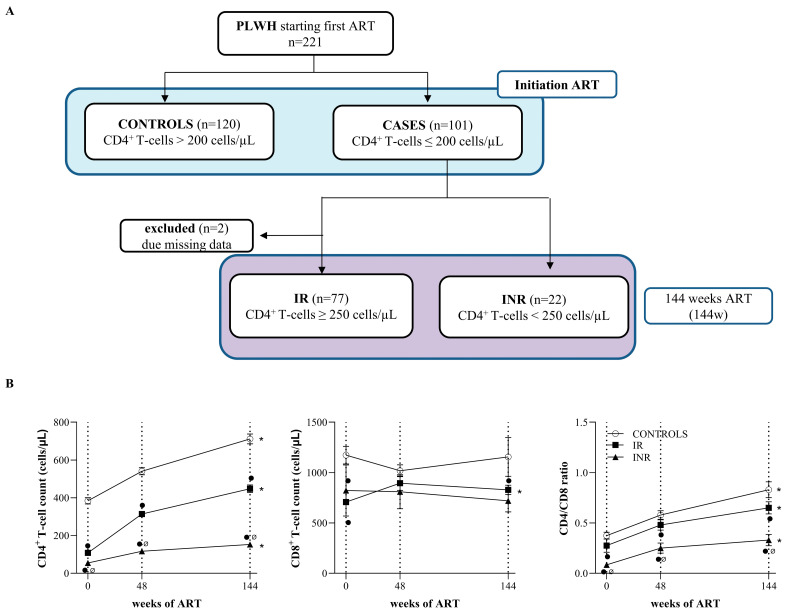
Study design. (**A**) Flow chart illustrating patient selection and enrolment. HIV-infected subjects were included and categorized as controls and cases according to the baseline ART threshold of 200 CD4^+^ T cell counts. Cases were categorized according to their immune status after 144 weeks of follow-up in “immunological recoverers” (IRs) or “immunological nonrecoverers” (INRs) depending on the threshold of CD4^+^ T cell counts of 250 CD4^+^ T cell counts/µL. (**B**) CD4 ^+^ T cell and CD8^+^ T cell count, and CD4/CD8 ratio in controls, IRs, and INRs during the study follow-up. * indicates significant differences at 144 w compared to baseline values using the Wilcoxon test. ● indicates significant differences compared to controls, and Ø indicates significant differences between INRs and IRs at each point of time, by nonparametric Mann–Whitney test.

The preART clinical characteristics of the study cohort are presented in Table 1. First, HIV-positive participants (*n* = 221) were categorized according to baseline CD4^+^ T cell counts (Figure 1A). Cases (baseline CD4^+^ T cell count ≤ 200 cells/µL) were older (*p* = 0.009), presented lower CD4^+^ and CD8^+^ T cell counts, and had a lower CD4/CD8 ratio (*p* < 0.001), but had a higher plasma viral load (VL) (*p* < 0.001) and prevalence of high blood pressure (HBP) (*p* = 0.043) than controls (baseline CD4^+^ T cell count > 200 cells/µL) at baseline. At 144 weeks, 21.8% of cases were classified as poor immune recoverers (INRs), whereas 76.2% of subjects showed good immunological reconstitution (IR). INRs presented a higher prevalence of HBP (*p* = 0.004).

PLHIV classified according to baseline CD4^+^ T cell counts as controls (baseline CD4^+^ T cell counts > 200 cells/µL) or cases (baseline CD4^+^ T cell counts ≤ 200 cells/µL). Cases were classified according to the absolute CD4^+^ T cell counts at 144 weeks of ART in immunological recoverers (IR, more or equal than 250 CD4^+^ T cell counts/µL) or immunological nonrecoverers (INR, less than 250 CD4^+^ T cell counts/µL).

As expected, on the basis of the classification criteria, we found that INRs had lower CD4^+^ T cell counts than IRs and controls at both 48 and 144 weeks of ART. The lower CD4^+^ T cell count in INRs was also accompanied by a lower CD4^+^/CD8^+^ ratio compared to IRs (*p* = 0.016 and *p* = 0.003 at 48 and 144 weeks, respectively) and controls (*p* < 0.001 at both 48 and 144 weeks). Both CD4^+^ T cell counts and CD4/CD8 ratio significantly increased in all groups as a consequence of ART adherence, whereas CD8^+^ T cell counts were only improved in IRs (*p* = 0.010) (Figure 1B).

### 2.2. Low APLNR and ZAG Concentrations Predicted Discordant Responses to ART

**Figure 2:**
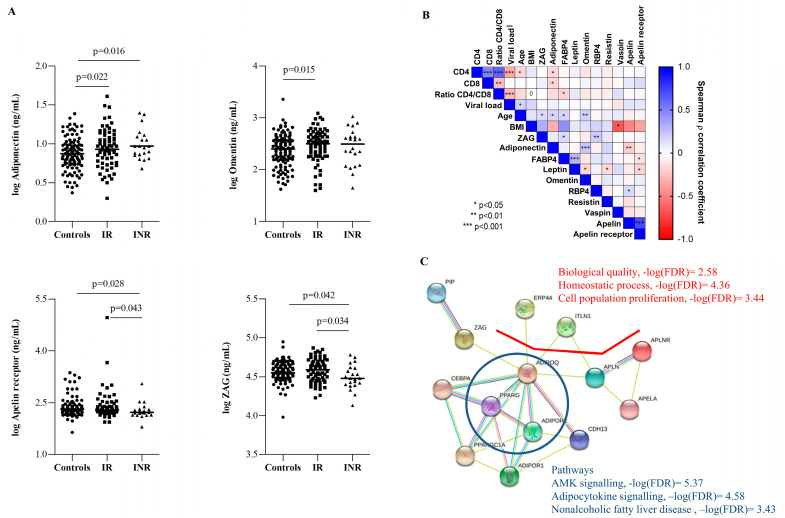
Circulating adipokine concentrations before initiating ART. (**A**) Baseline concentrations of adiponectin, omentin, APLNR, and ZAG in controls (*n* = 120), IRs (*n* = 77), and INRs (*n* = 22). *p*-value indicates significant differences among indicated groups by nonparametric Mann–Whitney test. (**B**) Heatmap showing the correlation analyses between all adipokines evaluated and clinical characteristics at baseline. Correlation matrix is color-coded according to the Spearman (ρ) correlation coefficient (−1:1, red: blue through white), and correlations with statistically significance are indicated. (**C**) Network interactions generated with the set of the four differentially expressed biomarkers at baseline (adiponectin, apelin receptor, omentin, and ZAG) identified by the STRING database. The STRING database identified different biological processes associated with our network using the false discovery rate (FDR). Adiponectin = ADIPOQ, apelin receptor = APLNR, omentin = ITLN1 and zinc-alpha-2-glycoprotein = ZAG.

Thus, according to the immune recovery status at 144 weeks, pre-ART circulating adiponectin concentrations were significantly higher in both IR and INR groups compared to controls (Figure 2A; *p* = 0.022 and *p* = 0.016, respectively), suggesting that adiponectin represents a marker of low nadir CD4^+^ T cell counts. On the other hand, circulating omentin concentrations were significantly higher in IR than in controls (*p* = 0.015). Interestingly, pre-ART circulating concentrations of APLNR and ZAG were significantly lower in INRs than in both controls and IRs (Figure 2A), suggesting that APLNR and ZAG are predictive markers of poor immune recovery independently related to a low nadir CD4^+^ T cell count. Multiple regression analyses for CD4^+^ T-cell reconstitution after adjusting for age, baseline plasma viral load, baseline CD4^+^ T cell count, and the ratio of CD4/CD8 showed that circulating APLNR concentrations were independently associated with CD4^+^ T-cell reconstitution (CD4^+^ T cell at 144 weeks, *p* = 0.041) (Supplemental Materials). The model (Model 1) was statistically significant (F(5119) = 19.806, *p* < 0.001) and accounted for approximately 40% of the variance of CD4^+^ T-cell reconstitution (R^2^ = 0.454, adjusted R^2^ = 0.431). In the case of ZAG, after adjusting for confounders, we found that low ZAG concentration was not an independent predictor for CD4^+^ T-cell reconstitution (*p* = 0.633) (Model 2). In fact, the model (Model 3) including both APLNR and ZAG concentration confirmed only APLNR as an independent predictor for CD4^+^ T cells at 144 weeks (*p* = 0.013). Concretely, low APLNR concentrations accounted for approximately 2% of the variance of CD4^+^ T cells at 144 weeks, as indexed by the squared semi-partial correlations.

Biomarker correlation analyses (Figure 2B) confirmed a negative correlation between both baseline CD4^+^ T cell and CD8^+^ T cell counts and adiponectin levels (ρ = −0.163, *p* = 0.017, and ρ = −0.172, *p* = 0.05, respectively), but positive correlations were observed between age and adiponectin (*p* < 0.05), omentin (*p* < 0.01), and ZAG concentrations (*p* < 0.05). Moreover, the correlation matrix confirmed a positive association between circulating adiponectin and omentin concentrations (*p* < 0.001). Curiously, both APLNR and ZAG concentrations were positively correlated with FABP4 concentrations (*p* < 0.05). In addition, the positive association of apelin receptor concentrations with circulating apelin concentrations was confirmed (*p* < 0.001), and ZAG concentrations were positively correlated with RBP4 concentrations (*p* < 0.01).

Then, metabolic pathways related to adiponectin, APLNR, omentin, and ZAG were evaluated (Figure 2C). The predicted interactions among the four biomarkers resulted in the enrichment of the response to several metabolic functions including response to stimulus and regulation of cellular process (−log(FDR) = 2.66 and −log(FDR) = 1.39, respectively), and may be related to identical protein-binding molecular functions (−log(FDR) = 2.60). Interestingly, APLNR and ZAG resulted in the enrichment of the regulation of biological quality, homeostatic process, and cell population proliferation (−log(FDR) = 2.58, −log(FDR) = 4.36, and −log(FDR) = 3.44, respectively). As expected, adiponectin was implicated in glucose homeostasis, lipid metabolic processes, and fatty acid oxidation (−log(FDR) = 4.36, −log(FDR) = 1.72, and −log(FDR) = 4.36, respectively). Hence, the predicted interaction between adiponectin and PPAR-γ may be related to the AMK signaling pathway (−log(FDR) = 5.37), adipocytokine signaling pathway (−log(FDR) = 4.58), and nonalcoholic fatty liver disease (−log(FDR) = 3.43).

### 2.3. APLNR and ZAG Concentrations Were Related to Poor Immune Progression

**Figure 3:**
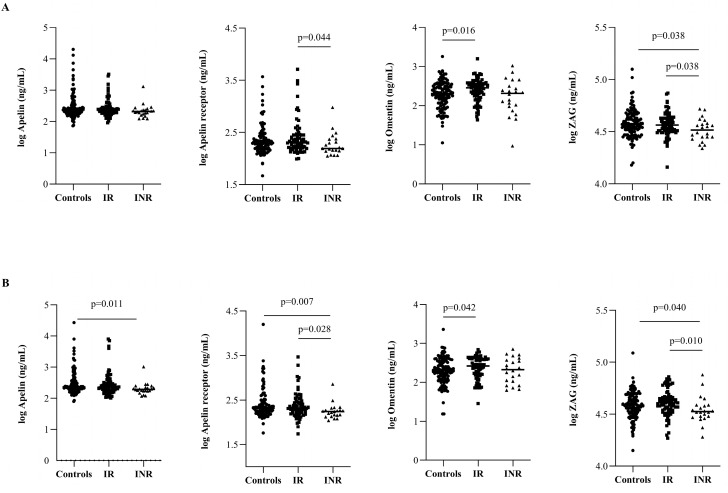
Follow-up circulating adipokine concentrations. Circulating apelin, APJ, omentin, and ZAG concentrations at (**A**) 48 weeks and (**B**) 144 weeks after ART in cases (IRs (*n* = 77) and INRs (*n* = 22)) compared to controls (*n* = 120). *p*-value indicates significant differences among groups by nonparametric Mann–Whitney test.

Circulating apelin, APLNR, and ZAG concentrations were significantly lower in INRs than in controls (*p* = 0.011 for apelin and *p* = 0.007 for APLNR at 144 weeks; *p* = 0.038 and *p* = 0.040 for ZAG at both 48 and 144 weeks, respectively) (Figure 3). In the case of IRs, circulating omentin concentrations remained higher at both 48 and 144 weeks (*p* = 0.016 and *p* = 0.042, respectively) than in controls. From 48 weeks onwards, circulating APLNR and ZAG concentrations were significantly lower in INRs than in IRs (*p* = 0.044 and *p* = 0.028 for APLNR, *p* = 0.038 and *p* = 0.01 for ZAG at 48 and 144 weeks, respectively).

### 2.4. APLNR and RBP4 Were Validated as Predictive Biomarkers of a Discordant Response

**Figure 4:**
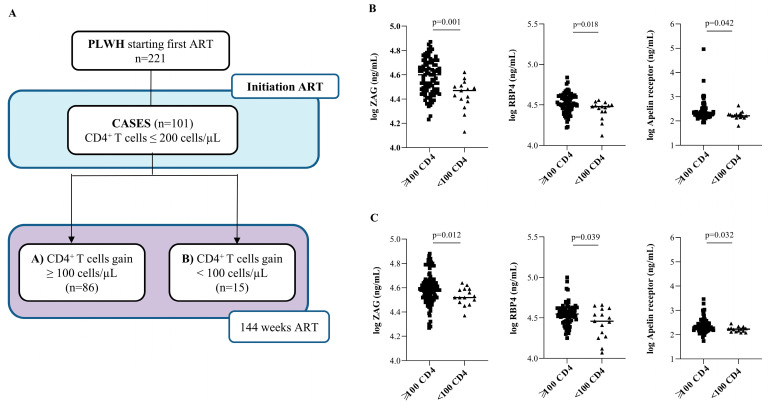
Validation cohort using CD4^+^ T-cell count gain as a marker of immune recovery. (**A**) Flow chart illustrating cases (*n* = 101) categorized according to CD4^+^ T-cell count gain after 144 weeks of ART initiation instead of 250 cell/µL CD4^+^ T-cell count cut-off: A group for those who gained ≥ 100 cells/µL, and B group for those who gained < 100 cells/µL. (**B**) Circulating ZAG, RBP4, and APLNR concentrations at baseline (**B**,**C**) at 144 weeks of ART. *p*-value indicates significant differences among indicated groups by nonparametric Mann–Whitney test.

To avoid a possible bias in the results due to the classification criteria previously selected (absolute number of CD4^+^ T cell count), we classified cases (*n* = 101) de novo into two groups according to the change in the CD4^+^ T cell count after 144 weeks of ART: A for individuals with a CD4^+^ T cell count gain ≥100 cells/µL after 144 weeks of ART or B for individuals with <100 CD4^+^ T cell count gain (Figure 4A). At baseline, the A group showed 99.11 ± 70.06 CD4^+^ T-cell counts/µL and a VL of 5.17 ± 0.66 log copies/mL compared to 87.33 ± 57.58 CD4^+^ T-cell counts/µL and a VL of 5.10 ± 0.66 log copies/mL in group B; these differences were not statistically significant. Patients with a worse immune response (B) showed significantly lower baseline ZAG, RBP4, and APLNR concentrations than patients with a more favorable response (A, CD4^+^ T cell gain ≥100) (*p* = 0.001, *p* = 0.018, and *p* = 0.042, respectively; Figure 4B). Interestingly, ZAG, RBP4, and APLNR concentrations remained significantly lower in patients who gained <100 CD4^+^ T cells/µL than those who gained ≥ 100 CD4^+^ T cells/µL (*p* = 0.012, *p* = 0.039, and *p* = 0.032, respectively) after 144 weeks of ART (Figure 4C). Multiple regression analyses for CD4^+^ T-cell reconstitution after adjusting for age, baseline plasma viral load, and baseline CD4^+^ T cell count was used to determine whether RBP4 concentrations were independently related with CD4^+^ T cells at 144 weeks in cases. The model (Model 1) was statistically significant (F(4,86) = 7.810, *p* < 0.001) and accounted for approximately 20% of the variance of CD4^+^ T-cell reconstitution (R^2^ = 0.266, adjusted R^2^ = 0.232). Concretely, circulating RBP4 concentrations accounted for approximately 4% of the variance of CD4^+^ T cells at 144 weeks, as indexed by the squared semi-partial correlations and APLNR concentration. Moreover, Model 2, including all adipokines, confirmed both low APLNR and RBP4 as independent predictors for CD4^+^ T cells at 144 weeks (*p* < 0.001). These results validated APLNR as predictive markers of poor immune recovery and suggested a potential role for RBP4 in the discordant response.

### 2.5. ART Affected FABP4, Omentin, and Resistin Concentrations

**Figure 5:**
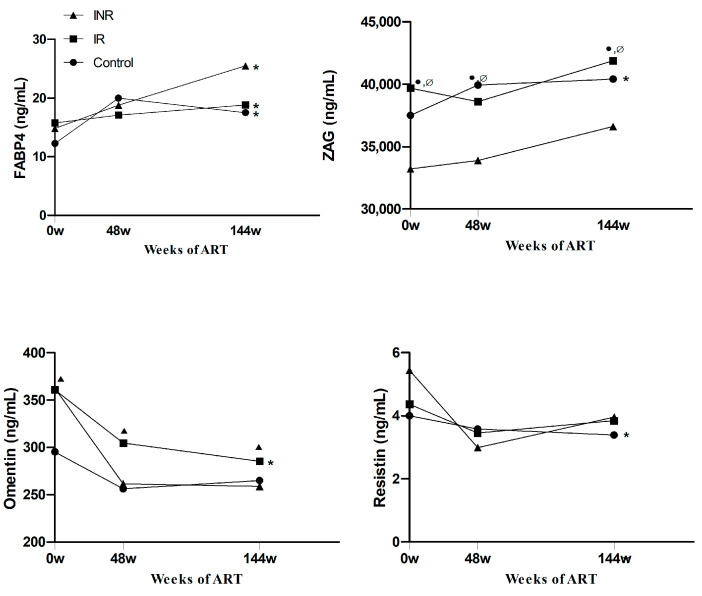
ART effect on circulating adipokine concentrations. Longitudinal study of circulating FABP4, ZAG, omentin, and resistin concentrations during ART follow-up. * indicates significant differences at 144 w compared to baseline values using the Wilcoxon test. ● indicates significant differences compared to controls (*n* = 120), ▲ indicates significant differences between controls and IR, and Ø indicates significant differences between INRs (*n* = 22) and IRs (*n* = 77) at each point of time, by nonparametric Mann–Whitney test.

For each group, the circulating concentration of each adipokine was included in follow-up progression curves (Figure 5). From baseline to 144 weeks, all groups exhibited increased circulating FABP4 concentrations, whereas the progressive increase in ZAG concentrations was only statistically significant in controls and IRs. Both controls and IRs also showed significant progressive increases in leptin, RBP4, and vaspin concentrations (data not shown). From baseline to 48 weeks, circulating omentin and resistin concentrations were significantly decreased in all groups. From 48 weeks to 144 weeks, circulating omentin concentrations were only significantly decreased in IRs, whereas circulating resistin concentrations decreased in both controls and IRs.

### 2.6. The Potential Effects of Different Antiretroviral Drugs on Adipokine Concentrations

**Figure 6:**
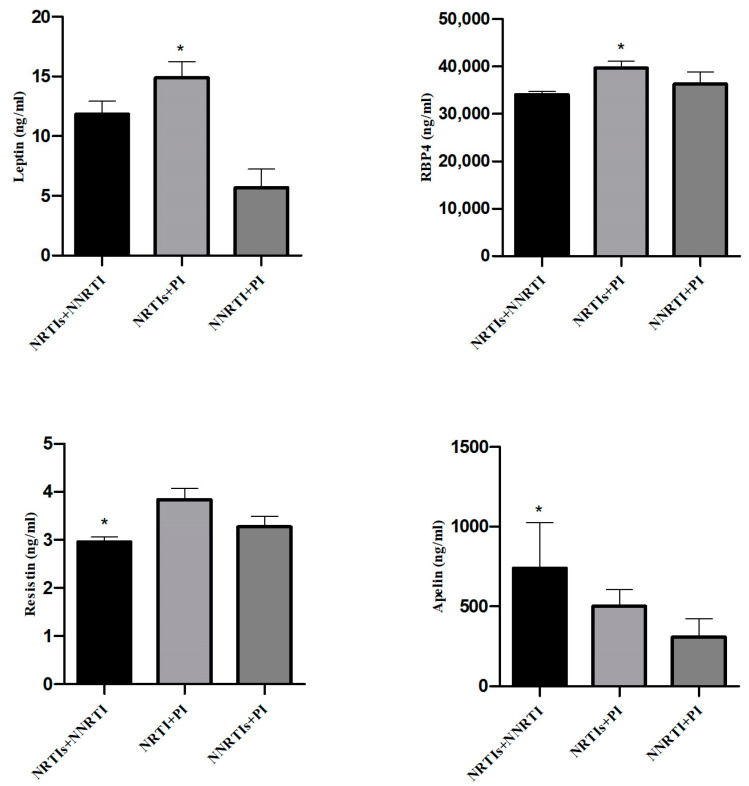
Adipokine concentrations in different ART schemes. (**A**) Circulating adipokine concentrations affected by the 2 NRTIs + PI scheme (*n* = 91) at 48 weeks. (**B**) Circulating adipokine concentrations affected by the 2 NRTIs + NNRTI scheme (*n* = 109) at 48 weeks (resistin) and 144 weeks (apelin). NNRTI + PI group included 8 patients. * indicates significant differences between ART schemes by nonparametric Kruskal–Wallis test.

Finally, the potential effect of the different antiretroviral therapies on circulating adipokine concentrations and CD4^+^ and CD8^+^ T-cell counts were evaluated (Figure 6). As expected, no differences were found in CD4^+^ and CD8^+^ T-cell counts or CD4^+^/CD8^+^ ratios when we compared the ART schemes during the follow-up (data not shown). Notably, after 48 weeks of treatment, circulating concentrations of leptin and RBP4 increased in subjects who received ART schemes containing two NRTIs plus a protease inhibitor (PI) (*p* = 0.018 and *p* = 0.005, respectively) (Figure 6A). PLHIV receiving ART schemes that contained two NRTIs + NNRTI presented lower resistin concentrations (*p* = 0.016) at 48 weeks and higher circulating apelin concentrations at 144 weeks (*p* = 0.009) (Figure 6B).

## 3. Discussion

Multiple and complex mechanisms are involved in the immunological recovery failure of PLHIV that achieve successful virological suppression. Although some factors that have been traditionally linked to poor immune recovery are being investigated [3,5,24,28], the complete mechanism preceding ART-associated poor immune recovery remains unclear. On the basis of previous results [10,12,29,30,31], we trust that adipokines may play a key role in immune reconstitution in PLHIV. Indeed, in this study, we postulated that low baseline circulating apelin receptor and ZAG concentrations were related to low CD4^+^ T-cell reconstitution (CD4^+^ T cell at 144 weeks) in PLHIV on ART and that their persistent low concentrations were related to poor immune progression. Low circulating RBP4 concentrations, which were positively associated with ZAG concentrations and related to the NNRTI ART scheme, were associated with a low CD4^+^ T cell gain. To our knowledge, this study is the first that has assessed the association of circulating adipokine concentrations with poor immune recovery in PLHIV that achieved successful virological suppression on ART.

A low CD4^+^ T-cell count (less than 200 cells/µL) and high VL in PLHIV initiating ART were strongly related to increased circulating adiponectin and omentin concentrations. Accordingly, low adiponectin levels have previously been related to a low VL and higher CD4^+^ T-cell counts in PLHIV [32,33]. Adiponectin is the richest adipokine found in the circulation [29], whereas omentin, also known as intelectin-1, is an adipokine abundantly present in the stromal vascular fraction of visceral fat depots [30]. Interestingly, both adiponectin and omentin seem to exert both anti-inflammatory and pro-inflammatory effects according to the cell-specific differences in the adiponectin- and omentin-induced secretory process. Indeed, on the one hand, both adiponectin and omentin induce macrophage differentiation towards the M2 phenotype [31] and inhibit the secretion of inflammatory cytokines, such as TNFα and IL-6 [34,35,36,37]. On the other hand, adiponectin and omentin promote the secretion of pro-inflammatory cytokines (e.g., IL-6 [38,39], IL-1, IFNγ [40]) and chemokines (e.g., MIP1α [39]) from activated CD4^+^ T cells [40] and stimulate the activation of inflammatory pathways, such as NF-kB signaling, or the shift towards the M1 phenotype. High circulating adiponectin levels are positively related to the severity of inflammation in individuals with autoimmune and chronic diseases, such as rheumatoid arthritis, chronic kidney disease, or inflammatory bowel disease [41].

Interestingly, circulating ZAG and apelin receptor concentrations perfectly discriminated poor immune recoverers (INRs) from both PLHIV starting ART with optimal CD4^+^ T-cell counts and PLHIV starting ART with low CD4^+^ T-cell counts who achieved a successful viral and immunological response to the therapy, suggesting that these adipokines are predictive factors of a discordant response in PLHIV. Indeed, both apelin receptor and ZAG concentrations remained significantly lower in INRs than in IRs at both 48 and 144 weeks of ART, which links the inhibition of these adipokines to a worse clinical prognosis of PLHIV.

ZAG is a novel adipokine that is expressed mainly in visceral and subcutaneous adipose tissue [42,43,44]. Information about its actions is scarce, but its role in the immune system is presumed to be mediated by anti-inflammatory effects on T cells and macrophages [45]. Notably, previous studies reported a negative correlation between ZAG, TNFα, and VCAM-1 concentrations [43]. The results from the present study indicated that untreated PLHIV with low CD4^+^ T-cell counts (less than 200 cells/µL) had a low-grade inflammatory background that was probably initiated by the activation of inflammatory pathways (such as NF-kB and IL1R) and traditional proinflammatory cytokines (such as IL6 and TNF), as well as the secretion of proinflammatory adipokines, such as adiponectin and omentin. Moreover, the release of the proinflammatory adipokines adiponectin and omentin into the circulation might negatively affect the expression of the anti-inflammatory ZAG protein via the activation of inflammatory pathways such as TNF signaling. Thus, the imbalance of circulating anti- and proinflammatory adipokines in the circulation of untreated and treated PLHIV could be crucial in the reconstitution of the CD4^+^ T-cell count and the subsequent immune response.

Apelin is an endogenous ligand of apelin receptor (APLNR), which is a G protein-coupled seven-transmembrane receptor [46] secreted by adipocytes [47] and is mainly known to be responsible for controlling cardiovascular function [48]. Interestingly, apelin inhibits the entrance of HIV into human cells by binding to APLNR, a coreceptor for T-tropic and dual tropic HIV-1 strains [46,49]. In this sense, both apelin and APLNR were negatively associated with the VL but positively correlated with the CD4^+^ T-cell count, which supports the relation of this adipokine and its receptor to HIV replication. Thus, the lower circulating apelin and apelin receptor concentrations in PLHIV with immune failure might denote a lack of inhibition of HIV entry into the cells and subsequently supports the connection between immune failure and persistent HIV reservoirs [50,51,52].

Regarding RBP4, although some evidence also confirmed the role of this adipokine in metabolic disturbance [53] in PLHIV, less is known about its potential role in the immune system. In PLHIV, higher levels of RBP4 were detected in those patients with metabolic syndrome (MetS), and a positive correlation between circulating RBP4 concentrations and the number of components of MetS was identified [54,55]. In another recent study, higher RBP4 levels were observed in frail HIV-infected people with VS [56].

The main limitation of this study is the lack of assessment of potential confounders for the measures of adipokine concentrations. Notably, the main objective of this study was to explore the role of adipokines in the immune system and not to study their relationship with metabolic syndrome. The group distribution is uneven, but from our perspective, this is a reflection of the epidemiological reality. The percentage of INRs in the population ranges from 10–40% [2], and thus our classification adjusts to this percentage (27.7%).

## 4. Materials and Methods

### 4.1. Study Design and Participants

This multicenter, longitudinal case–control study included 221 adult treatment-naïve HIV-infected subjects who were consecutively recruited between 2011 and 2013 at the HIV outpatient clinic of the participating hospitals and who started their first ART and achieved virological suppression after ART. Patients were selected from among those who were receiving a combination of two nucleoside reverse transcriptase inhibitors (NRTIs) plus a nonnucleoside reverse transcriptase inhibitor (NNRTI) or a protease inhibitor (PI) or 2 NNRTIs plus a PI. A flow chart of the patient selection and enrolment process is provided in Figure 1. Of the selected subjects, 120 were considered controls (baseline CD4^+^ T-cell counts >200 cells/μL), and 101 were cases (baseline CD4^+^ T-cell counts ≤ 200 cells/μL). At 144 weeks, cases were classified according to the cut-off of 250 CD4^+^ T cells [2,57]. Among the cases, 77 subjects (76.2%) achieved a value of more than 250 CD4^+^ T cells/µL after 144 weeks of ART (“immunological recoverers”, IR), whereas 22 subjects (21.8%) did not reach the 250 CD4^+^ T cells/µL threshold (“immunological nonrecoverers”, INRs). Two subjects included in the case group at baseline were excluded due to missing values. Thus, from the 221 subjects initially included in the study, 219 subjects achieved the 144 weeks follow-up being classified as 120 controls, 77 IRs, and 22 INRs.

All the selected patients were required to fulfil the following inclusion criteria: aged ≥ 18 years, presence of HIV-1 infection, on an ART regimen during the 48 weeks of the study, and undetectable plasma HIV-1 viral load (VL) at 48 weeks. The exclusion criteria were the presence of active opportunistic infections, current inflammatory diseases or conditions, changes in the ART regimen, and the use of drugs known to affect/modify CD4^+^ T-cell counts (antineoplastic drugs, steroids, immune response modulators, and colony-stimulating factors, among others).

### 4.2. General Laboratory Measurements

Blood was drawn from a peripheral vein after an overnight fast. Whole blood was used to determine the CD4^+^ T-cell count and for DNA isolation. HIV-1 infection was diagnosed by a positive ELISA and confirmed by Western blot analysis. The plasma HIV-1 VL was determined using the Cobas Amplicor HIV-1 Monitor Test v 1.5 (Roche Diagnostics, Barcelona, Spain). The limit of detection was <20 copies/µL. CD4^+^ T-cell counts were analyzed using a FAC flow cytometer FAC (Becton Dickinson, San Jose, CA, USA). These determinations were carried out in a central laboratory. Plasma was obtained by centrifugation and stored at −80 °C at BioBanc IISPV until use.

### 4.3. Adipokine Measurements

Plasma concentrations of human adiponectin (RD195023100, BioVendor, Brno, Czech Republic), apelin (E00237, QAYEE, Shanghai, China), apelin receptor (E05144, QAYEE, Shanghai, China), fatty acid-binding protein 4 (FABP4) (RD191036200R, BioVendor, Brno, Czech Republic), leptin (EL2001-1, AssayPro, St Charles, MO, USA), omentin (RD191100200R, BioVendor, Brno, Czech Republic), retinol-binding protein 4 (RBP4) (ER3005-1, AssayPro, St Charles, MO, USA), resistin (RD 191016100, BioVendor, Brno, Czech Republic), vaspin (RD191097200R, BioVendor, Brno, Czech Republic), and zinc-alpha-2-glycoprotein (ZAG) (RD191093100R, BioVendor, Brno, Czech Republic) were measured using a one-step double-antibody sandwich enzyme-linked immunosorbent assay (ELISA) according to the manufacturer’s instructions at baseline, as well as at 48 and 144 weeks of ART follow-up.

### 4.4. Statistical Analyses

Before the statistical analyses, the normal distribution and homogeneity of the variances were tested using a Kolmogorov–Smirnov test. Normally distributed data are presented as the means ± standard deviations (SD), whereas variables with a skewed distribution are presented as the medians (25th percentiles–75th percentiles) or were transformed into a decimal logarithm. Categorical variables are reported as numbers (percentages). Qualitative variables were analyzed using the χ^2^ test or Fisher’s exact test when necessary. Comparisons between groups were performed with nonparametric Kruskal–Wallis (KW) and/or Mann–Whitney (MW) tests for unpaired samples and a Wilcoxon *t*-test for paired samples (W). When applicable, the Bonferroni post hoc test was used for multiple comparisons. Stepwise multiple linear regression analyses were used to determine whether adipokines were independently related with CD4^+^ T-cell reconstitution (CD4^+^ T cell at 144 weeks was used as dependent variable). Correlation analyses were evaluated using the Spearman test. Statistical analyses were performed using SPSS (version 21.0, SPSS Inc., Chicago, IL, USA), and graphical representations were generated with GraphPad Prism software (version 9.0, GraphPad Inc., San Diego, CA, USA). The results were considered significant at *p* < 0.05.

## 5. Conclusions

Therefore, in conclusion, adipokines are essential factors that contribute to the immune response to HIV infection. Although some adipokines function as proinflammatory molecules and others as anti-inflammatory molecules, a proper balance is needed to maintain a minimum inflammatory environment and might be crucial for the reconstitution of CD4^+^ T-cell count and to promote a good recovery status in PLHIV. To the best of our knowledge, this study is the first to appoint a specific role to some anti-inflammatory and proinflammatory adipokines in the immune response of PLHIV with poor immune recovery. Interestingly, low circulating apelin receptor and RBP4 and ZAG concentrations were related to low CD4^+^ T-cell reconstitution (CD4^+^ T cell at 144 weeks) in PLHIV. Concretely, apelin receptor and RBP4 concentrations resulted in being independently related to CD4^+^ T-cell counts at 144 weeks of successful suppressive antiretroviral therapy, making apelin receptor and RBP4 potential biomarkers of a discordant response in untreated PLHIV. Further investigations are needed to elucidate the mechanisms by which these adipokines modulate in the immune response.

## Figures and Tables

**Table 1:** Study cohort baseline characteristics in ART-naïve PLHIV according to baseline CD4^+^ T cell counts (controls vs. cases) and CD4^+^ T cell counts at 144 weeks of ART (IR vs. INR).

	Controls (*n* = 120, 53.8%)	Cases (*n* = 101, 46.2%)	*p* Value * (Controls vs. Cases)	IR (*n* = 77, 76.2%)	INR (*n* = 22, 21.8%)	*p* Value ** (IR vs. INR)
**Demographic** **characteristics**						
Age (years)	38 (31–45)	41 (35–51)	**0.009**	40 (35–50)	44 (41–56)	0.063
Male (%)	101 (84.2)	80 (79.2)	0.383	61 (79.22)	16 (72.72)	0.765
BMI	24.72 ± 2.93	24.07 ± 3.92	0.535	24.07 ± 3.91	24.09 ± 3.85	0.615
CD4^+^ T-cell count (cells/mm^3^)	328 (260–465)	88 (31–169)	**<0.001**	116 (43–174.5)	38 (12.5–70)	**0.003**
CD8^+^ T-cell count (cells/mm^3^)	1069 (780–1341)	527 (357–1020)	**<0.001**	521 (359–1058)	436 (222–1015)	0.536
CD4^+^/CD8^+^ ratio	0.28 (0.21–0.49)	0.12 (0.06–0.34)	**<0.001**	0.14 (0.08–0.37)	0.05 (0.028–0.12)	**0.010**
Alcohol (%)	26 (21.7)	20 (19.8)	0.746	13 (16.88)	6 (27.27)	0.345
Tobacco (%)	51 (42.5)	33 (32.7)	0.070	28 (36.36)	5 (22.72)	0.394
Comorbidities						
High blood pressure (%)	6 (5)	13 (12.9)	**0.043**	8 (10.39)	4 (18.18)	**0.004**
Dyslipidaemia (%)	0	1 (1)	0.484	1 (1.29)	0	0.590
Diabetes mellitus (%)	10 (8.3)	8 (7.9)	0.764	4 (5.19)	3 (13.63)	0.093
**HIV-related parameters**						
Viral Load (log copies/mL)	4.79 (4.21–5.17)	5.29 (4.72–5.61)	**<0.001**	5.28 (4.73–5.57)	5.39 (4.71–5.71)	0.695
Risk factor			0.492			0.185
Heterosexual (%)	33 (27.5)	38 (37.6)		31 (40.26)	6 (27.27)	
Homosexual (%)	58 (48.3)	45 (44.6)		35 (45.45)	9 (40.91)	
IVDU (%)	9 (7.5)	8 (7.9)		7 (9.09)	1 (4.54)	
Haemophilic (%)	1 (0.8)	1 (1)		0	1 (4.54)	
Unknown/others (%)	19 (15.8)	10 (9.9)		4 (5.19)	5 (22.72)	
HIV-Coinfections						
HBV (%)	37 (30.8)	36 (35.6)	0.778	28 (36.36)	6 (27.27)	0.601
HCV (%)	17 (14.2)	13 (12.9)	0.938	9 (11.69)	4 (18.18)	0.343
HAART						
NRTI+NNRTI (%)	54 (45)	55 (54.5)	0.225	40 (51.95)	12 (54.54)	0.830
NRTI+PI (%)	54 (45)	37 (36.6)	0.218	29 (37.66)	8 (36.36)	0.912
NNRTI+PI (%)	4 (3.3)	4 (3.9)	1	3 (3.90)	1 (4.54)	0.892
Unknown/other (%)	8 (6.7)	6 (5.9)	1	5 (6.49)	1 (4.54)	0.222

Data are presented as n (%) media ± SD or median (interquartile range). Categorical data were compared through a χ^2^ test, whereas continuous data were compared using nonparametric Mann–Whitney test (*p* value * for comparison between controls and cases, *p* value ** for comparison between IRs and INRs). *p*-value < 0.05 was considered significant and is highlighted in bold.

## Data Availability

The data that support the finding of this study are available from the corresponding author upon reasonable request.

## Supplementary Materials

The following supporting information can be downloaded at: https://www.mdpi.com/article/10.3390/ijms23042202/s1.
