# Supplementary material for: Adipokines as New Biomarkers of Immune Recovery: Apelin Receptor, RBP4 and ZAG Are Related to CD4+ T-Cell Reconstitution in PLHIV on Suppressive Antiretroviral Therapy
Source: Int J Mol Sci. 2022 Feb 17;23(4):2202. doi: 10.3390/ijms23042202 (PMC8874604; doi:10.3390/ijms23042202)
Supplement: Supplementary file 1 [file ijms-23-02202-s001.zip › ijms-1575467-supplementary(1).pdf]

Article

Adipokines as new biomarkers of immune recovery: Apelin receptor, RBP4 and ZAG are related to $CD4^+$ T cell reconstitution in PLHIV on Suppressive Antiretroviral Therapy

Elena Yeregui [1,2,3,14], Jenifer Masip [1,3,14], Consuelo Viladés [1,2,3,14], Pere Domingo [4], Yolanda M. Pacheco [5,6], Julià Blanco [7,8,11,14], Josep Mallolas [9,14], Verónica Alba [1,3,14], Montserrat Vargas [1,14], Graciano García-Pardo [1,2,3,14], Eugènia Negredo [10,11,14], Montserrat Olona [1,3,14], Judit Vidal-González [12], Maria Peraire [3], Anna Martí [1,2,14], Fréderic Gómez-Bertomeu [1,3,14], Manuel Leal [13], Francesc Vidal [1,2,3,14,*], Joaquim Peraire [1,2,3,14,ϕ] and Anna Rull [1,2,3,14,ϕ,*]

1 Hospital Universitari de Tarragona Joan XXIII (HJ23), Tarragona, Spain
2 Institut Investigació Sanitària Pere Virgili (IISPV), Tarragona, Spain
3 Universitat Rovira i Virgili (URV), Tarragona, Spain
4 Infectious Diseases Unit, Hospital de la Santa Creu i Sant Pau, Barcelona, Spain
5 Laboratory of Immunology, Institute of Biomedicine of Seville, IBiS, Seville, Spain
6 UGC Clinical Laboratories, Virgen del Rocío University Hospital/CSIC/University of Seville, Seville, Spain
7 IrsiCaixa AIDS Research Institute, Badalona, Spain
8 Germans Trias i Pujol Research Institute (IGTP), Can Ruti Campus, Badalona, Spain
9 HIV Unit and Infectious Diseases Service, Hospital Clinic-IDIBAPS, Barcelona, Spain
10 Fundació de la Lluita contra les Infeccions, Hospital Universitari Germans Trias i Pujol, Badalona, Spain
11 Universitat de Vic-Universitat Central de Catalunya (UVic-UCC), Vic, Spain
12 Universitat de Barcelona (UB), Barcelona, Spain
13 Internal medicine Service, Hospital Viamed Santa Ángela de la Cruz, Seville, Spain
14 CIBER Enfermedades Infecciosas (CIBERINFEC), Instituto de Salud Carlos III, Madrid, Spain

ϕ These authors contributed equally to this paper, and should both be considered as senior co-authors.
*Correspondence: fvidalmarsal.hj23.ics@gencat.cat (F.V.); anna.rull@iispv.cat (A.R.)

Supplemental Table S1. Stepwise regression results to determine whether AR and ZAG were independently related to CD4+ T-cell at 144 weeks.

	Model	B	SE-b	Beta	Pearson r	sr^2
Model 1 Apelin receptor	Constant	113.391	171.240			
	Age*	-3.814	1.919	-0.142	-0.242	0.018
	Viral Load*	62.968	30.679	0.145	0.031	0.019
	CD4+ T-cell*	1.075	0.126	0.624	0.626	0.335
	Ratio CD4/CD8 baseline	11.645	58.310	0.015	0.222	0.001
	AR baseline*	0.089	0.043	0.143	0.149	0.019
Model 2 ZAG	Constant	180.451	170.059			
	Age*	-4.338	1.871	-0.164	-0.238	0.024
	Viral Load*	65.188	29.199	0.154	0.026	0.022
	CD4+ T-cell*	1.094	0.126	0.641	0.634	0.340
	Ratio CD4/CD8 basal	-10.911	57.093	-0.014	0.213	0.001
	ZAG baseline	-0.001	0.002	-0.033	0.058	0.001
Model 3 Apelin receptor +ZAG	Constant	176.764	135.801			
	Age*	-3.738	1.497	-0.148	-0.263	0.019
	Viral Load*	50.352	23.175	0.132	-0.115	0.014
	CD4+ T-cell*	1.028	0.092	0.678	0.656	0.391
	ZAG baseline	7.699E-05	0.001	0.003	0.042	-
	AR baseline*	0.092	0.037	0.142	0.154	0.019

The dependent variable was $CD4^+$ T cell counts at 144 weeks.

sr^2 is the squared semi-partial correlation; *p<0.05

Model 1: $F(5,119) = 19.806$, $p < 0.001$, R2=0.454, Adjusted R2=0.431

Model 2: $F(5,124) = 19.844$, $p < 0.001$, R2=0.444, Adjusted R2=0.422

Model 3: $F(5,164) = 31.549$, $p < 0.001$, R2=0.490, Adjusted R2=0.475

Supplemental Table S2. Stepwise regression results to determine whether RBP4 was independently related to CD4+ T-cell at 144 weeks .

	Model	B	SE-b	Beta	Pearson r	sr^2
Model 1 RBP4	Constant	-1.532	158.506			
	Age*	-3.596	1.544	-0.216	-0.182	0.046
	Viral Load*	52.844	23.701	0.215	0.079	0.042
	CD4+ T-cell baseline*	1.276	0.264	0.469	0.380	0.199
	RBP4 baseline*	0.004	0.002	0.211	0.151	0.043
Model 2 Apelin receptor+ RBP4+ZAG	Constant	-150.190	174.376			
	Age*	-4.197	1.569	-0.263	-0.206	0.061
	Viral Load*	69.504	27.713	0.242	0.133	0.053
	CD4+ T-cell*	1.225	0.260	0.447	0.374	0.190
	RBP4 baseline*	0.004	0.002	0.205	0.203	0.038
	Apelin receptor baseline*	0.072	0.034	0.199	0.272	0.037
	ZAG baseline	0.002	0.001	0.134	0.063	0.015

The dependent variable was $CD4^+$ T cell counts at 144 weeks.

sr^2 is the squared semi-partial correlation; *p<0.05

Model 1: $F(4,86) = 7.810$, $p < 0.001$, R2=0.266, Adjusted R2=0.232

Model 2: $F(6,75) = 6.970$, $p < 0.001$, R2=0.358, Adjusted R2=0.307
